# Fluorescent Carbon Quantum Dots—Synthesis, Functionalization and Sensing Application in Food Analysis

**DOI:** 10.3390/nano10050930

**Published:** 2020-05-11

**Authors:** Mingfei Pan, Xiaoqian Xie, Kaixin Liu, Jingying Yang, Liping Hong, Shuo Wang

**Affiliations:** 1 State Key Laboratory of Food Nutrition and Safety, Tianjin University of Science and Technology, Tianjin 300457, China; panmf2012@tust.edu.cn (M.P.); qianxx8135@163.com (X.X.); lkx13642168374@163.com (K.L.); yangjy0823@126.com (J.Y.); honglpstu@163.com (L.H.); 2 Key Laboratory of Food Nutrition and Safety, Ministry of Education of China, Tianjin University of Science and Technology, Tianjin 300457, China

**Keywords:** CQDs, synthesis, fluorescent sensing, food analysis

## Abstract

Carbon quantum dots (CQDs) with stable physicochemical properties are one of the emerging carbon nanomaterials that have been studied in recent years. In addition to the excellent optical properties such as photoluminescence, photobleaching resistance and light stability, this material also has favorable advantages of good biocompatibility and easy functionalization, which make it an ideal raw material for constructing sensing equipment. In addition, CQDs can combined with other kinds of materials to form the nanostructured composites with unique properties, which provides new insights and ideas for the research of many fields. In the field of food analysis, emerging CQDs have been deeply studied in food composition analysis, detection and monitoring trace harmful substances and made remarkable research progress. This article introduces and compares the various methods for CQDs preparation and reviews its related sensing applications as a new material in food components analysis and food safety inspection in recent years. It is expected to provide a significant guidance for the further study of CQDs in the field of food analysis and detection.

## 1. Introduction

Carbon quantum dots (CQDs), also called carbon dots (CDs), were a kind of zero-dimensional nanomaterial with size less than 10 nm, which were first discovered in 2004 [1,2,3]. As a sort of environmental-friendly carbon nanomaterial [4,5], CQDs possess stable physicochemical properties and have good biocompatibility and ability to disperse in water phase [6,7,8]. They are easy to be prepared and functionalized [9]. CQDs with quasi-spherical microstructure have excellent optical properties including controllable and stable fluorescent characteristics, resistance to photobleaching capacity and excellent ultraviolet (UV) absorption ability [10,11,12,13], having attracted considerable attention of the researchers and become a new research hotspot in the field of materials. Generally, according to the different carbon cores, CQDs are usually divided into graphene quantum dots (GQDs), carbon nanodots (CNDs) and polymer dots (PDs) [14]. No matter which type of CQDs, they all have very diminutive particle size and large specific surface area. The surface atoms have a high activity and are liable to connect with other atoms or chemical groups to achieve different purposes [15]. Compared with conventional semiconductor quantum dots (QDs) and organic fluorescent dyes [16,17], CQDs not only have preferable optical properties but also reduce the participation of toxic metal elements in the preparation process, thus reducing the adverse impact on the environment and realizing low-cost and green synthesis [18,19]. So far, CQDs with different properties based on various raw materials or methods have been developed for different applications, which demonstrated the favorable application prospects in the numerous fields of medicine, chemistry, food and environment [20,21].

For food analysis, the instrumental detection strategies based on the principles of chromatography and mass spectrometry such as high performance liquid chromatography (HPLC), gas chromatography (GC), gas chromatography-mass spectrometry (GC-MS), liquid chromatography-mass spectrometry (LC-MS) are highly efficient and accurate, which can almost cover the analysis and detection of multifarious targets including functional components, the residues of pesticides and veterinary drugs, heavy metal ions, mycotoxins, illegal additives and so forth [22,23,24]. However, this kind of methods usually requires relatively expensive large-scale analytical instruments and complicated sample preparation processes, having a gap in detection speed, real-time and on-site analysis. In the past few years, the emerging sensing analysis is equipped with the merits of high sensitivity, good accuracy, low cost, simplicity and convenience, which has drawn increasingly the attention of researchers [25,26]. The green-synthesized CQDs possess high fluorescent intensity, good stability and resistance to photobleaching capacity, having significant advantages in fluorescent sensing analysis [27,28]. The controlled synthesis of CQDs with different properties can be achieved using different sources of carbon and nitrogen by combination with different synthesis methods. As a fluorescent sensing probe, CQDs have a very wide application prospect in the analysis and detection of functional compositions and trace harmful substances in foods.

This paper has reviewed the different methods of CQDs preparation and the existing research findings of recent five years in the field of food analysis in order to explore the applicability and practical value of CQDs as a new nanomaterial in fluorescent sensing and biomimetic analysis, providing relevant references for further research in the field of food analysis.

## 2. Various Strategies of CQDs Synthesis

In recent years, great progress has been made in the preparation of CQDs. Depending on the carbon source used, the synthesis methods of CQDs can generally be divided into top-down and bottom-up approaches [29,30]. The top-down method is mostly used in the early stage of research and usually prepares CQDs by stripping and cutting large-size carbon materials with physical or chemical ways; and the bottom-up method aggregates small organic molecule into CQDs by means of chemistry. These two approaches can meet the different requirements of small particle size and excellent optical properties of CQDs to some extent [31,32].

### 2.1. Top-Down Approach

In the top-down approach, physical or chemical processes are often applied to strip and cut large-sized carbon materials to obtain small-sized CQDs, which are more common in the early stages of CQDs research. As early as 2004 and 2005, Xu and Bottini obtained CQDs with different quantum yields (QY) by arc discharge treatment of single wall/multi wall carbon nanotubes (SWCNT, MWCNT), respectively [33,34]. At present, carbon-based materials with large particle size such as graphite, carbon nanotubes and activated carbon have been used for the preparation of nanosized CQDs. And the treatment method has also been extended to arc, laser, chemical reagent or high potential methods [35,36,37]. However, this kind of methods are usually carried out under the conditions of high acidity, high potential and high energy, which are relatively difficult to control. Consequently, there are scarce reports in the synthesis of CQDs in recent years.

**Figure 1:**
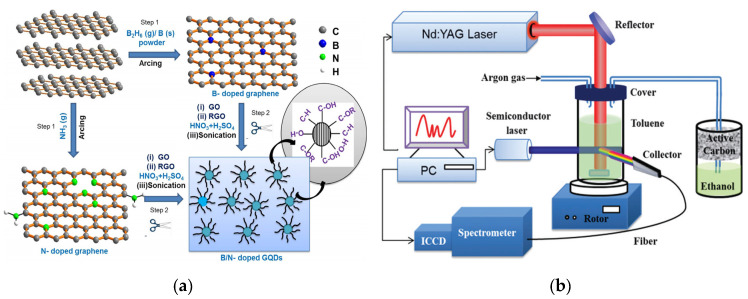
(**a**) Schematic of the synthesis of B/N-carbon quantum dots (CQDs) (Step1: synthesis of doped graphene by arc discharge; Step2: chemical shearing process of graphene sheets). Reproduced with permission from [40]. Copyright Chemical Physics Letters, 2014; (**b**) Schematic illustration of the device of laser ablation synthesis of CQDs. Reproduced with permission from [43]. Copyright Chemical Communications, 2016.

Although arc discharge was first used in the synthesis of CQDs, the QY, size and uniformity of the prepared CQDs are insufficient. Few-layered graphene QDs (GQDs, a type of CQDs) has similar size, synthesis routes, photoluminescence (PL) behavior and physical/chemical properties to CQDs [38,39]. Usually, other atoms or materials can be introduced to improve the performance of CQDs. Dey and the co-workers synthesized B, N-doped GQDs (B/N-GQDs) with size of 4–6 nm by arc discharge of graphite electrodes and chemical shearing in the atmosphere of H_2_ + He + B_2_H_6_ and H_2_ + He + NH_3_ mixtures, respectively [40] (Figure 1a). Both the GQDs and B/N-GQDs have strong blue emission related to excitation wavelength-independent. However, the introduced B and N atoms substitute some of the C atoms in the arcing process of graphite, resulting in a small blue-shift in the PL emission of N-GQDs compared to GQDs. Biazar et al. have reported a process of synthesizing CQDs and CQDs/TiO_2_ composite by direct-current arc discharge between graphite electrodes with high-purity [41]. The introduced TiO_2_ plays an important role in enhancing the photocatalytic activity of CQDs and slowing down the electron-hole recombination rate. Under the catalysis of visible light, the prepared CQDs/TiO_2_ composite shows stronger applicability than TiO_2_.

High-energy laser can also be used to etch carbon materials to obtain CQDs with definite fluorescent QY. Sun et al. first reported a synthesis method for CQDs using laser etching a carbon target in water vapor and combined with subsequent acid treatment to make CQDs have bright PL and a small size, being beneficial to obtain higher QY [42]. Yu synthesized CQDs by irradiating toluene with non-focusing pulsed laser, in which the key step is further ablation of intermediate graphene. Under the adjustable intensity of the input laser, controllable synthesis of CQDs with different optical properties can be realized [43]. Figure 1b has showed a schematic illustration of a device for real-time monitoring the fluorescent change of the product, which provides a new method for controllable synthesis of fluorescent materials. Compared with the single pulse laser [44], the advantage of the double pulse laser technology lies in the use of the shock wave generated by the second pulse to further ablate the generated CQDs to obtain smaller particle size. This method can form abundant functional groups on the surface of CQDs and enhance the optical and catalytic sensing performance. Nguyen et al. prepared CQDs with size as low as 1 nm using this method [45]. Laser etching is one of the effective physical methods for the synthesis of CQDs with narrow size distribution, good water solubility and prominent fluorescent characteristic. However, this method requires rigorous experimental conditions, complicated devices and post-processing steps, thus presenting lower common applicability in the practical production.

**Figure 2:**
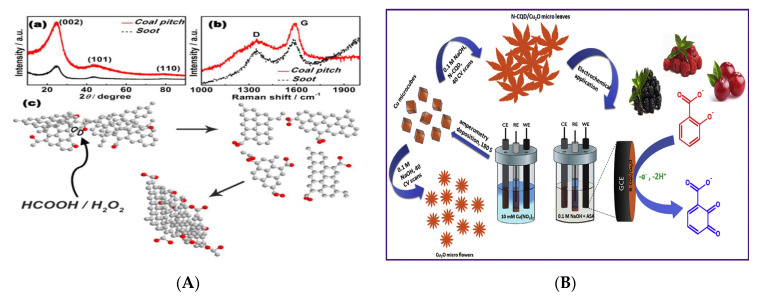
(**A**) Schematic of synthesis and characterization of CQDs by chemical oxidation (a: X-ray diffraction (XRD) patterns; b: Raman spectra; c: formation schematic of CQDs). Reproduced with permission from [48]. Copyright Chemical Communications, 2017; (**B**) Schematic of electrochemical preparation of N-CQDs/Cu_2_O and ASA detection in berries. Reproduced with permission from [54]. Copyright Journal of Colloid and Interface Science, 2018.

Chemical oxidation is one method for CQDs synthesis that treats carbon materials using strong oxidants (nitric acid, sulfuric acid and hydrogen peroxide). This method is easy to operate, fast and highly repeatable and provides the possibility for large-scale production of CQDs. Liu et al. first synthesized fluorescent CQDs with QY of 0.8–1.9% and diameter less than 2 nm by chemical oxidation [46]. It is worth noting that the adjustable fluorescent of CQDs in this study provides a new strategy for synthesizing multicolor luminous CQDs. Chen et al. treated citric acid and polyethyleneimine (PEI) by refluxing and heating and obtained PEI-CQDs with a size distribution of 1–6 nm, which was used for the detection of Cu^2+^ and H_2_S based on inner filter effect (IFE) [47]. The advantage of the refluxing method is that CQDs with larger emission wavelength and uniform particle size distribution can be obtained without tedious passivation and purification steps. Under the conditions without additional heating and energy input, Meng and the co-workers synthesized different CQDs (Size: 3–5 nm, QY: 5.4–10.1%) using the chemical reaction of coal pitch powder with formic acid and H_2_O_2_ [48] (Figure 2a). The CQDs yield of 49% was achieved in this research, providing a reliable foundation for mass production of CQDs. By controlling the crystallization time and molecular weight screening, Bao et al. prepared CQDs with different sizes and oxidation degrees of surface state via exfoliating carbon fiber fragments with the reflux of nitric acid [49]. The fluorescent-emission of the prepared CQDs covered the part of visible spectrum from blue to red based on the narrowing of energy band, thus facilitating diversified applications of multi-color imaging. In the process of chemical oxidation, the chemical reagents used are highly corrosive and require strict experimental environments. Therefore, finding a mild oxidant is an important research direction for the preparation of CQDs by chemical oxidation.

Based on the principle of redox of conductive working electrode in electrolyte, the electrochemical synthesis of CQDs can be accomplished under the normal temperature and pressure. The modification of the hydrophilic groups (−OH, −COOH, −NH_2_, etc.) on the surface of CQDs can be achieved by controlling the electrolyte components and the electrochemical oxidation-reduction process [50,51]. Different emission spectra usually require different excitation wavelengths but it is a severe challenge to obtain and preserve the white-light spectrum [52]. Joseph and his co-workers successfully synthesized the white-light CQDs with QY of 11.51% through electrochemical reaction between double graphite rod electrodes in the battery structure and further applied in light-emitting devices in illuminating systems [53]. The white luminescence of CQDs derives from broad emission spectrum and the heterogeneity of size and functional groups. Compared with other reports, this study has great breakthroughs on the mechanism of fluorescent QY and illuminant color. Muthusankar et al. synthesized the N-CQDs and N-CQDs/Cu_2_O composite by electrochemical deposition and applied for sensitive and selective detection of non-steroidal anti-inflammatory drug aspirin (ASA) in berries [54] (Figure 2b). In this study, the size-controllable CQDs improves the stability of Cu_2_O and the electron transfer rate without affecting its crystallinity. In terms of the results of electrode modification, the composite of N-CQDs/Cu_2_O has better electrochemical oxidation performance than N-CQDs and Cu_2_O. This N-CQDs/Cu_2_O composite also has distinct advantages in stability, reproducibility and response sensitivity, providing an effective sensing strategy for the design of new functional CQDs probes. According to the current researches, electrochemical synthesis is one of the most studied and commonly used top-down methods for preparing CQDs. However, in the preparing process with high-potential electrolytes, the experimental conditions are difficult to control and the purification process is relatively complicated, which is an urgent problem to be solved.

In general, the methods of top-down mainly appear in the early research of CQDs synthesis, because the carbon precursors are relatively expensive and the post-processing processes are complicated, which limit the application of this kind of methods [55,56,57]. However, CQDs prepared by such high-energy methods have a good sp^2^ conjugate structure, which facilitates the expression of intrinsic luminescence from the carbon core. Although the sizes and properties of CQDs are difficult to control in these methods, the CQDs with different optical properties and quantum efficiency can still be obtained by optimizing the reaction conditions, reducing the reaction speed and refining the subsequent purification procedures [58].

### 2.2. Bottom-Up Approach

In the bottom-up approach, the energy of microwave, hydrothermal and ultrasound is usually applied to aggregate small organic molecules or oligomer precursors to synthesize nanometer-sized CQDs [59,60]. Under the environment of high radiation, high heat and high frequency, the generated CQDs can be equipped with both high QY and excellent optical properties. This kind of method has been favored by researchers in the preparation of CQDs in recent years, because the preparation and operation are simple, reaction conditions are controllable and raw materials are inexpensive, which provide the possibility for the one-step high-volume synthesis of CQDs.

#### 2.2.1. Microwave-Assisted Method

In the synthesis of CQDs, microwave with homogeneous energy is widely applied due to the superiority of rapidity, high efficiency and convenience. As early as 2009, Zhu and his co-workers first synthesized fluorescent CQDs with a narrow size distribution by the pyrolysis of polyethylene glycol and saccharide under high frequency microwave [61]. In 2017, Choi et al. pyrolyzed the AB_2_ type lysine utilizing microwave-induced thermal polyamidation and carbonization and obtained the water-soluble CQDs with QY of 23.3% [62]. This synthesis process can be completed in 5 min. This research has demonstrated that the branching points in the precursor are the critical factor to the CQDs production with high mass yield and fluorescent QY, having offered a new direction for the rapid preparation of CQDs with excellent performances.

**Figure 3:**
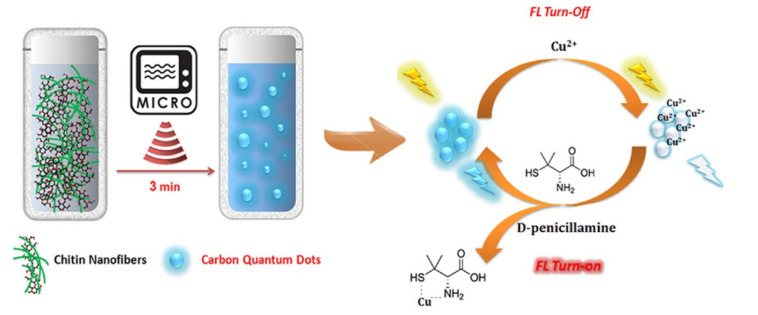
Schematic of the process of microwave-assisted synthesis of CQDs and “on-off” transformation of CQDs-Cu^2+^. Reproduced with permission from [63]. Copyright Journal of Industrial and Engineering Chemistry, 2017.

Figure 3 shows the synthesis process of CQDs (Size: 2–10 nm, QY: 5.1%) from chitin nanofibers using microwave assisted-hydrothermal method in 3 min and “on-off” transformation of CQDs-Cu^2+^ for the drug sensing based on quenching effect [63]. The fabricated CQDs exhibits high stability and sensitive and selective fluorescent to *D*-penicillamine. Zhang et al. synthesized N, S doped CQDs (NS-CQDs) with an average diameter of 3.3 nm by microwave-assisted treatment of vitamin C and thiourea, which had a red shift in the wavelength range of 280–400 nm, resulting from the electromagnetic waves formed by the surface functional groups [64]. Compared with ordinary CQDs, the doped atoms not only adjust the surface energy and electron energy of CQDs but also improve the physical and chemical properties of CQDs and its selectivity to the target molecules [65,66]. By optimizing the experimental conditions and comparing the cross-linking degree of different hydroxy compounds (propanol, *1,2*-propanediol, glycerin), Feng et al. finally obtained N-CQDs (Size: 2.8 nm, QY: 27.9%) by microwave heating of *2*-azidazole and glycerin [67]. This research verified the close correlation of fluorescent QY and chemical natures of cross-linked internal core, which offered the theoretical foundation to design adjustable luminous probes with high QY. Based on the static quenching effect, the prepared N-CQDs was further successfully used as a “turn-off” fluorescent nanoprobe for the detection of Ag^+^. Judging from the previous reports, microwave heating is a fascinating and promising tool for the preparation of carbon materials, especially for CQDs with small-size [68,69,70]. The approach of microwave-assisted has become one of the commonly used and indispensable methods of the CQDs synthesis because of the characteristics of time and energy saving, controllable operation, no requirement for complex equipment and so on.

#### 2.2.2. Ultrasound-Assisted Method

**Figure 4:**
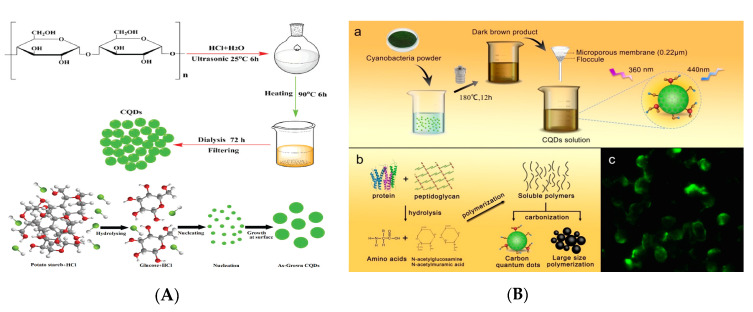
(**A**) Schematic of the ultrasound-assisted synthesis and the formation mechanism of CQDs. Reproduced with permission from [72]. Copyright New Journal of Chemistry, 2019; (**B**) Schematic of the hydrothermal synthesis of CQDs (a: synthesis routes of CQDs; b: formation mechanism of CQDs; c: fluorescent cell imaging). Reproduced with permission from [73]. Copyright Polymers, 2019.

In 2011, Li and his co-workers first ultrasonically treated the glucose in an acid or base environment and successfully obtained PL CQDs with diameter of less than 5 nm and QY of 7% [71]. This ultrasound-assisted method for CQDs synthesis employs the high energy of ultrasonic wave to crack carbon materials into nanoparticles (NPs) at the presence of acid, alkali or oxidant, which is considered as a new pathway of CQDs synthesis. The use of high energy of ultrasonic wave avoids the complex post-treatment process, thereby realizing the facile synthesis of CQDs with small size. Figure 4a has illustrated the ultrasound-assisted synthesis process and the formation mechanism of CQDs with green luminescence from potato starch [72]. This prepared CQDs acts as an effective fluorescent sensing probe for sensitive and selective detection of Zn^2+^ in aqueous solution. The oxygen-rich groups on the surface of CQDs can be modified using other materials, which has further significant applications in the aspects of sensing and catalysis.

Huang has reported an ultrasound-assisted synthesis strategy of a thiol-terminated polyethylene glycol (PEG-SH) functionalized fluorescent CQDs [74]. By comparing the morphology and performance of CQDs and PEG-SH-CQDs, it has demonstrated that the introduction of hydrophilic PEG not only improves its dispersibility in aqueous phase but also endows the CQDs with excellent biocompatibility, which has remarkable significance for the fabrication of CQDs with designable properties and functions. Lu et al. synthesized a blue fluorescent N-CQDs (Size: 2.5–5.5 nm, QY: 3.6%, Average life: 3.02 ns) by ultrasonic treatment of dopamine in dimethylformamide (DMF), which exhibited good stability of colloid and light in aqueous solution [75]. Compared with the CQDs (Size: 2–10 nm) prepared by Huang, even if the same solvent (DMF) is used in the synthesis process, the obtained sizes of the CQDs are different, which verifies the importance of the carbon source used in the preparation process. From the current research progress, the ultrasound-assisted method has the characteristics of low requirements for equipment, simple operation and time saving but the QY of CQDs is usually low, which is difficult to meet the requirements of high fluorescent performance. Therefore, it is necessary to control the preparation process strictly.

#### 2.2.3. Hydrothermal Method

Hydrothermal method is another bottom-up strategy under high temperature and pressure for the synthesis of CQDs, in which uses small organic molecules including glucose, fructose, amino acids and other natural products as the precursors. In 2010, Zhang et al. reported the synthesis of CQDs (Size: 2 nm, QY: 6.75%) using a one-step method by hydrothermal treatment of ascorbic acid, which was the first report on hydrothermal synthesis of fluorescent CQDs [76]. It has demonstrated that the PL efficiency and fluorescent QY of CQDs are closely associated with their size and shape, the used solvent and the reaction time in hydrothermal process. Another biomass-based CQDs (Size: 2.48 nm, QY: 9.24%) from the single carbon source cyanobacteria was prepared by Wang using hydrothermal method and successfully applied as the fluorescent probe for cell imaging due to the bright luminescence [73] (Figure 4b). These results have verified the synthesis process of the prepared CQDs without the participation of chemical reagents, which provides a sustainable synthesis strategy for further research.

The strategies of surface functionalization and heteroatom doping play a crucial role in regulating the surface properties and fluorescent intensity of CQDs and improving the recognition selectivity of CQDs to the targets [77,78]. As an effective artificial recognition material, molecularly imprinted polymers (MIPs) have made great contributions to the enhancement of specificity and selectivity of biomimetic sensing detection. Sun et al. treated mango peels by hydrothermal method to obtain the fluorescent CQDs and applied as a fluorescent sensing probe in the MIP composite (CQDs@MIPs) [79]. After the removal of template, CQDs@MIPs can selectively detect targets through the specific interaction between the target and the binding sites, which provides an effective strategy for the recognition and capture of trace target in complex matrix. On the other hand, the improvement of the performances of CQDs with high QY can arise from the doping of heteroatoms in the synthesis process of CQDs [80,81]. In a polytetrafluoroethylene-lined autoclave at 180 °C, Zhao et al. synthesized the homogeneous N-CQDs with an average diameter of 3.3 nm and a QY of 23.1% [82]; Liu et al. treated the ammonium citrate and betaine hydrochloride under high temperature (200 °C) and pressure for 5 h and obtained monodisperse spherical N-CQDs (Size: 5–8 nm; QY: 46.01%) [83]. By comparison of these results, although the same fluorescent N-CQDs is obtained, the QY and size are different which can be attributed to the sources of carbon and nitrogen and the insufficiency of reaction degree. Depending on the activation energy trap effect related to abundant surface functional groups, the fluorescent intensity of N-CQDs is dependent of the excitation wavelengths [84]. The PL behavior of the highly luminous N-CQDs is independent of the emission wavelength, which avoids the needless auto-fluorescent, potentially showing a broad prospect in bioimaging and biosensing. The hydrothermal method with remarkable characteristics of simple operation, environmental friendliness, low energy-consumption and cost has been one frequently-used method of CQDs synthesis. However, this method requires high reaction temperature and long reaction time, causing insufficient reaction of some raw materials during the reaction process, which needs to be improved.

## 3. Sensing Applications of Functionalized CQDs in Food Analysis

With the continuous deepening of researches on related techniques, CQDs with different performances have been synthesized and functionalized and then successfully applied in many research fields and especially in the sensing analysis, which have made remarkable research progress [85,86]. In terms of food analysis, CQDs with excellent performances have been used as sensing probes in food components (such as phenolic compounds, saccharides, vitamins, proteins, amino acids, etc.) or harmful substances (such as pesticides and veterinary drugs residues, illegal additives, heavy metals, mycotoxins, etc.) [87,88,89]. This type of studies not only deepens the basic theoretical research of CQDs but also expands the related applications of CQDs. It also provides new strategies for the analysis of components in food and the rapid detection of trace harmful substances.

### 3.1. Functional Components in Foods

In the field of food analysis, CQDs acts as a sensing probe to detect and analyze functional components of food such as protein, vitamins, phenolic compounds and so on, which not only has excellent optical properties but also has significant advantages of pro-environment, low price, convenience and speediness. Foods can provide enough energy and nutrients for human body, such as protein, fat, carbohydrates, vitamins and minerals, which are essential for normal life activities. As a result, the detection of nutrients in foods is very necessary.

**Figure 5:**
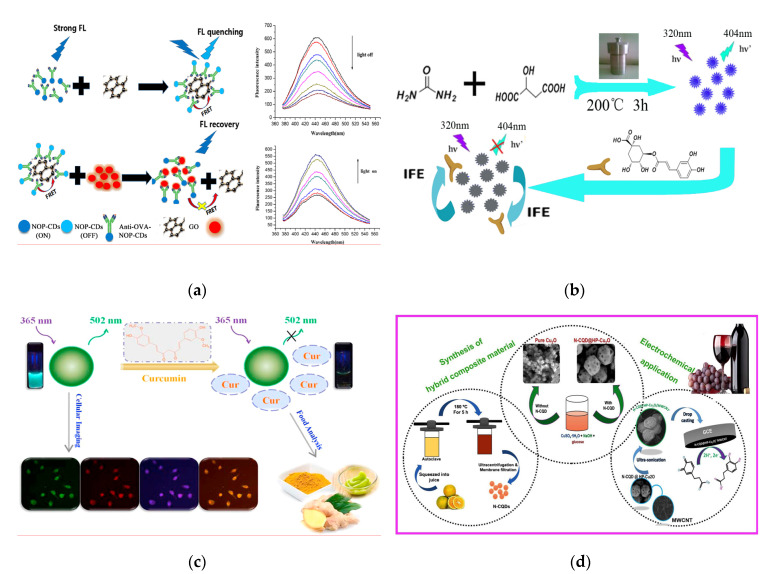
(**a**) Schematic of the quantitative detection for ovalbumin (OVA) based on fluorescent resonance energy transfer (FRET) of N, O, P co-doped CQDs (NOP-CQDs). Reproduced with permission from [91]. Copyright Sensors and Actuators B: Chemical, 2018; (**b**) Schematic of the detection of chlorogenic acid in honeysuckle using CQDs. Reproduced with permission from [95]. Copyright Spectrochimica Acta Part A-molecular and Biomolecular Spectroscopy, 2018; (**c**) Schematic of the quenching mechanism of Cur to NP-CQDs and the applications of cellular imaging. Reproduced with permission from [101]. Copyright Talanta, 2018; (**d**) Schematic of the synthesis pathway of the composite (CQDs@HP-Cu_2_O/MWCNT) composed of CQDs, hexagonal porous Cu_2_O (HP-Cu_2_O) and multi-walled carbon nanotubes (MWCNT) and caffeic acid detection process. Reproduced with permission from [103]. Copyright Composites Part B-Engineering, 2019.

The content of ovalbumin (OVA) is deemed as a reference for evaluating the quality of protein [90]. Fu et al. synthesized a novel CQDs co-doped with N, O, P (NOP-CQDs) through one-step hydrothermal method and applied for quantitative detection of OVA in the egg products [91] (Figure 5a). In the fluorescent resonance energy transfer (FRET) system composed of NOP-CQDs, graphene oxide and anti-OVA, the “on-off” sensing probe has achieved the selective recognition and capture of OVA based on the specific interaction of antigen-antibody, which achieved a limit of detection (LOD) of 153 µg L^−1^. Purbia et al. developed a highly luminescent CQDs (Size: 1–6 nm) with green and blue fluorescent for detecting vitamin B_1_ in commercial vitamin capsule (LOD: 280 nmol L^−1^) [92]. The detection principle is that the gradual addition of vitamin B_1_ can restore the fluorescent of CQDs quenched by Cu^2+^, thereby achieving the rapid detection of targeted component. Certain small molecular substances present in plant-derived foods have specific antibacterial, anti-inflammatory or anti-oxidant properties, which gives the foods special medicinal properties. These small molecules are defined as “functional components” and this kind of foods is called “medicine-homologous foods” [93,94]. Qualitative or quantitative analysis of the functional components in medicinal-homologous foods is usually the main method to evaluate their quality. Fluorescent CQDs provides an effective, convenient and accurate strategy for the analysis and detection of such efficacy components. Chlorogenic acid has remarkable antibacterial and antiviral pharmacological effects, the amount of which is the main basis for evaluating the quality of honeysuckle (a medicinal-homologous food). Yang et al. synthesized the water-soluble CQDs (Size: 2.1 nm, QY: 16.5%) via hydrothermal treatment of malic acid and urea and applied for the fluorescent sensing detection of chlorogenic acid in honeysuckle [95] (Figure 5b). Due to the mechanism of IFE [96], the fluorescent of CQDs is effectively quenched as the concentration of chlorogenic acid increases in the linear range of 0.15–60 µmol L^−1^, with a lower LOD of 45 nmol L^−1^. By comparison with the HPLC and HPLC-MS/MS for chlorogenic acid detection [97,98], the developed CQDs-based method not only has good improvement on the sensitivity but also significantly improves the detection speed, which is suitable for rapid screening of large quantities of honeysuckle samples. Curcumin (Cur) is an acidic polyphenolic substance extracted from the roots of ginger plants and its main chain is unsaturated aliphatic and aromatic groups. As a yellow pigment, it is often used as a meat coloring agent and acid-base indicator and has anti-inflammatory, antioxidant and other pharmacological effects [99,100]. Liu et al. have rapidly synthesized one N and P dual-doped CQDs (NP-CQDs) using glucose as carbon source, *1,2*-ethylenediamine as N-dopant and concentrated phosphoric acid as P-dopant, which was further utilized as a label-free sensor for Cur determination [101] (Figure 5c), achieving a linear range of 0.5–20 µmol L^−1^ and a LOD of 58 nmol L^−1^. In practical samples (drinking water and foods), satisfactory relative standard deviations (RSD) and recoveries were 0.08–5.39% and 95.2–105.2%, respectively. Additionally, this NP-CQDs can be used as effective fluorescent agent for cellular imaging without noticeable cytotoxicity.

Based on the luminescent characteristic of CQDs and the selective adsorption of MIPs, CQDs-embedded MIPs has provided new methods for the fluorescent analysis of trace substances in complex food matrices. Using the metal organic frameworks (MOFs) as the core of surface molecular imprinting, Xu et al. designed a novel nanocomposite of CQDs@MOF@MIP and further developed a fluorescent sensor for the detection of quercetin (QCT) in extract capsule of Ginkgo biloba. The fluorescent sensor showed remarkable sensitivity and selectivity to QCT in the wide concentration range of 0–50.0 µmol L^−1^ with a LOD of 2.9 nmol L^−1^ (S/N = 3) [102]. This CQDs@MOF@MIP sensing model has high specific surface area and ample cavities and further possesses the ability of signal amplification and conversion, which can transform chemical signal into the detectable fluorescent signal by binding with target molecules, potentially becoming an innovative technology. Figure 5d has shown the synthesis process of the composite composed of the N-CQDs decorated hexagonal porous Cu_2_O and MWCNT and the application of N-CQDs@HP-Cu_2_O/MWCNT for the detection of caffeic acid (CA) in red wine [103]. The fabricated fluorescent sensing device was demonstrated to possess high sensitivity, good repeatability and stability to CA. The doped CQDs and conductive MWCNT make the composite have higher specific surface area and porosity and further improve the electrocatalytic activity of Cu_2_O-based materials [104,105]. This study provides a strong guidance for the fabrication of various porous nanocomposites. Rutin is a flavonol glycoside widely present in plants and can be used as an edible antioxidant and nutrition enhancer. Sinduja et al. synthesized CQDs (Size: 7 nm) using the non-essential amino acid asparagine as a precursor and further exploited it for the determination of rutin by spectrofluorimetry based on the decrease in emission intensity at 441 nm [106]. Good linear relationship (R^2^ = 0.997) was obtained in the range of 0.5–15 µmol L^−1^ with a LOD of 0.1 µmol L^−1^. In this study, the intrinsic fluorescent characteristic of CQDs and the selective π-π interaction between rutin and the CQDs aromatic rings enhance the detection accuracy and reliability to the target.

### 3.2. Poisonous and Harmful Substances in Foods

#### 3.2.1. Pesticide and Veterinary Drug Residues

The residues of pesticides and veterinary drugs in foods, including the drug prototypes and their metabolites that accumulate in foods, are potentially harmful to human health [107]. The effective analysis to these residues is one important topic in the research of food safety. The emerging CQDs material can offer more accurate, efficient and cost-effective fluorescent sensing, which has made great progress in the detection of pesticides and veterinary drugs residues in food in recent years [108,109].

The MIPs with specific recognition ability are commonly used for the purification of complex matrices and the enrichment and separation of trace target molecules of pesticides and veterinary drugs in foods. The emerging CQDs-embedded MIPs have provided a very meaningful strategy of the detection of small molecule harmful substances in foods. Zhang and the co-workers have successfully designed a room-temperature ionic-liquid (RTIL)-sensitized CQDs sensing probe (RTIL-SCQDs-MIPs) and applied for sensitive detection of insecticide lambda-cyhalothrin (LC) in vegetable and tea samples, with a LOD of 0.5 µg kg^−1^ [110]. It has demonstrated that the introduced S-doped CQDs and RTIL in the sensing probe can enhance the performances of recognition and sensing. Based on the mechanism of host-guest interactions [111], the outer MIPs-based material plays an important role in the identification of target LC in tested samples, which also provides a very promising prospect for the specific identification of trace targets in complex food matrices. This type of composites has combined the advantages of a single material with different functions to form a synergistic enhancement effect and provides a very interesting strategy in the detection of small molecules.

**Figure 6:**
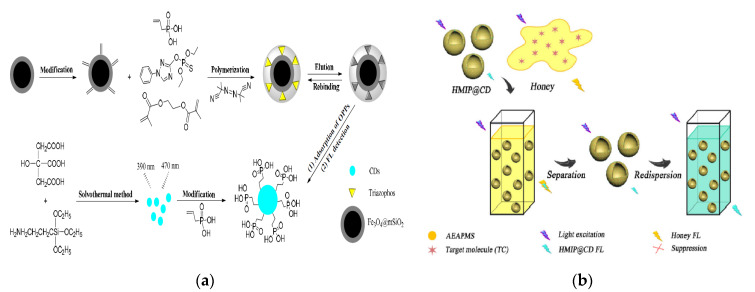
(**a**) Schematic of the preparation of core-shell magnetic molecularly-imprinted microspheres (MMIPs) of vinyl phosphate (VPA)-functionalized CQDs (MMIPs-CQDs@VPA) and the detection process for triazophos. Reproduced with permission from [112]. Copyright Polymers, 2019; (**b**) Schematic of fluorescent detection process of tetracycline (TC) in honey. Reproduced with permission from [115]. Copyright Talanta, 2018.

Wu et al. have developed a vinyl phosphate (VPA)-functionalized, magnetic MIP (MMIP) microspheres for the enrichment of organophosphorus pesticide residues and further combined with CQDs for fluorescent detection [112] (Figure 6a). Dual functional monomers and mesoporous SiO_2_-modified Fe_3_O_4_ magnetic particles (Fe_3_O_4_@mSiO_2_) have prominent advantages in the formation of recognition cavities in MIPs, which further improve the sensing properties of the hybrid composites. This MMIP-CQDs@VPA fluorescent sensor was further applied for the detection of triazophos in cucumbers with a lower LOD of 0.0015 mmol L^−1^, which was proved to possess high accuracy, good sensitivity and repeatability. Fu et al. have constructed one fluorescent sensor based the composite of CQDs and Fe^3+^ which has good response to ampicillin in mineral water, milk and pork samples with a LOD of 0.7 µmol L^−1^ [113]. This merit of the research benefits by the binding sites provided by the surface functional groups of CQDs for the metal ion Fe^3+^ and this sensor offers more feasible and promising method in the simultaneous detection of multiple targets. Compared to the nano-optical sensor based on the core-shell polypyrrole-CdTe QDs-MIP [114], the CQDs/Fe^3+^-based sensor did not need the complicated synthesis process and long reaction time. Importantly, the use of CQDs to take place of heavy metals-based QDs has reduced the potential toxicity in practical applications. Li et al. fabricated the single-hole hollow MIP-CQDs fluorescent sensor (HMIP@CQDs) using sol-gel method for sensitive and rapid detection of tetracycline (TC) in honey samples [115] (Figure 6b). In this study, HMIP was employed as a recognition element for the targeted recognition and fluorescent detection of TC (LOD: 3.1 µg L^−1^, Recovery: 93–105%). The HMIP@CQDs showed better fluorescent performance than the traditional solid core MIP@CQDs [116]. Mahmoud and the co-workers have designed an electrochemical sensor based on the composite MIP-AuNPs/NS@GQDs, which has good sensitivity for detecting antiviral drug sofosbuvir [117]. Due to the successful electro-polymerization of NS@GQDs and AuNPs on pencil graphite electrodes, good conductivity and electrocatalytic activity were obtained in the electrochemical sensing process.

**Figure 7:**
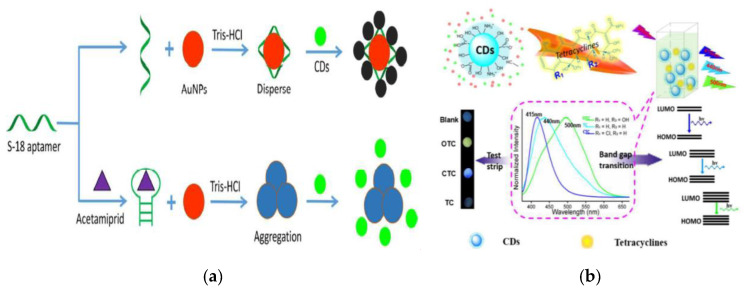
(**a**) Schematic of the fluorescent aptasensor for acetamiprid detection based on AuNPs and CQDs. Reproduced with permission from [121]. Copyright Analyst, 2018; (**b**) Schematic of three TCs for visual detection using CQDs-based test strips. Reproduced with permission from [124]. Copyright Nanoscale, 2018.

Noble metal NPs, such as AuNPs and AgNPs, have a large specific surface area and low energy transfer resistance and are an ideal carrier of electrochemical sensing [118,119,120]. The composite materials with CQDs provide a better sensing interface for electrochemical and fluorescent sensing, which can achieve higher sensitivity and accuracy in the detection of harmful substances in foods. A novel fluorescent aptasensor composed of AuNPs and CQDs was fabricated for sensitively and selectively detecting acetamiprid pesticides in tomato, cucumber and cabbage sample [121] (Figure 7a). One aptamer S-18 (recognition element) was introduced to combine with AuNPs to effectively quench the fluorescent of CQDs to achieve the detection of target (LOD: 1.08 µg L^−1^), which provides a new idea for the construction of specific and functionalized fluorescent sensors. An electrochemical sensing platform was constructed using the composite of Ag/Ag_2_O@NS-CQDs for the detection of catechol, achieving a low LOD of 13 nmol L^−1^ [122]. It has demonstrated that the Ag/Ag_2_O@NS-CQDs composite has better conductivity, specific surface area and electrocatalytic ability than that of NS-CQDs and its participation greatly increases the electrochemical activity on the sensing interface, which has a remarkable guiding significance for the construction of various electrochemical sensors with excellent performance.

With the development of fluorescent immunosensor, the emerging CQDs has provided new ideas for the fabrication of economic and convenient immunoassay, potentially becoming an alternative and green fluorescent reagent [123]. Miao et al. reported the synthesis process of CQDs with blue fluorescent and its application as a sensing probe in the visual recognition and quantitative detection of three TCs [124] (Figure 7b). In this study, the LODs for TC, oxytetracycline (OTC) and chlortetracycline (CTC) are 5.18 nmol L^−1^, 6.06 nmol L^−1^ and 14 nmol L^−1^ respectively, which are lower than the obtained results in the above report [115]. The fluorescent signals of the three TC targets on the CQDs-based test strip are obviously different and can be distinguished visually, so that multiple targets in the one sample can be detected synchronously and sensitively.

#### 3.2.2. Heavy Metal Ions

**Figure 8:**
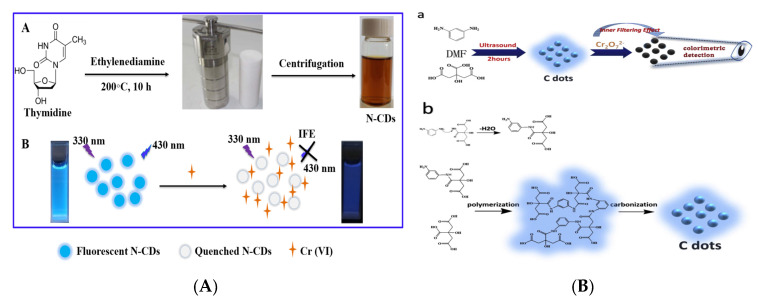
(**A**) Schematic of the detection of Cr^6+^ by N-CQDs fluorescent sensor. Reproduced with permission from [129]. Copyright Spectrochimica Acta Part A-molecular and Biomolecular Spectroscopy, 2019; (**B**) Schematic of the CQDs for the detection of Cr_2_O_7_^2−^ (a: the detection mechanism of Cr_2_O_7_^2−^; b: the synthesis route of CQDs). Reproduced with permission from [131]. Copyright Dyes and Pigments, 2019.

Heavy metal ions such as Fe^3+^, Cu^2+^, Fe^2+^, Ag^+^, Cr^6+^, Au^3+^, Hg^2+^ are an important aspect that cause the pollution of water or environment, which are easy to accumulate in animals and plants [125]. After entering the human body through the food chain, they can produce accumulated toxicity. It is of great significance to develop an effective, convenient and sensitive method for detecting heavy metal ions in foods [126,127,128]. Ming et al. synthesized a simple and low-cost N-CQDs (QY = 47.5%) via one-step hydrothermal method using thymidine as carbon source to fabricate a fluorescent sensor for sensitive detection of Cr^6+^ [129] (Figure 8a). Through the IFE, the obtained N-CQDs exhibited good fluorescent response to the target Cr^6+^. A good logarithm correlation between the fluorescent intensity of N-CQDs and the concentration of Cr^6+^ was obtained in the range of 0.1–430 µmol L^−1^ with R^2^ of 0.992 and low LOD of 1.26 nmol L^−1^. The fluorescent sensor can finish the detection process less than 1 min and has good repeatability, reproducibility and stability. Similarly, Lu et al. constructed a unique fluorescent 3D paper-based analysis device for Cr^6+^, in which applied blue N-CQDs as the fluorescent substrate [130]. In both above studies, the fluorescent quenching mechanism based on the IFE was used in the construction of different detection devices.

Figure 8b has shown a dual-mode (fluorometric and colorimetric) CQDs fluorescent nano-sensing onsite platform for the detection of Cr_2_O_7_^2−^ in drinking water [131]. The water-soluble CQDs was prepared using a simple one-pot ultrasonic irradiation method, which had the excitation-dependent feature that the achieved emission evolved from blue (440 nm) to green luminescence (528 nm). The CQDs can be remarkably quenched by the chromate due to IFE and possesses highly selective and sensitive responses to Cr_2_O_7_^2−^ through the color changes at the same time. The dual sensing mode has established an effective assay for the recognition and detection of Cr_2_O_7_^2−^ with LODs as low as 0.14 nmol L^−^^1^ and 410 nmol L^−^^1^, respectively.

The CQDs-based test strips have obvious advantages in time-consuming and testing costs, which have a very broad development space in terms of rapid, sensitive and low-cost testing. One highly luminescent N-CQDs was synthesized by Raji et al. using jackfruit seeds as carbon source by a facile, green and rapid one-step microwave-assisted method, which displayed excellent water solubility, high QY (17.91%) and photo-stability, longer storage stability (stable up to >180 days without agglomeration) and low cytotoxicity [132]. The PL intensity of the resulting N-CQDs was linearly, selectively and sensitively quenched by Au^3+^ ions and the LOD of 239 nmol L^−1^ was obtained. Han et al. established a novel CQDs-based strategy for the ratiometric fluorescent detection of Cu^2+^ and glutathione (GSH) [133]. The fluorescent CQDs was firstly obtained through one-pot facile hydrothermal treatment using *o*-phenylenediamine (OPD) and citric acid as precursors. The oxidation product *2,3*-diaminophenazine was obtained through the oxidation reaction of OPD and Cu^2+^, can not only emerge a new emission peak at 562 nm but also quench the fluorescent of CQDs at 446 nm (maximum emission) through FRET [134]. This ratiometric sensing system showed higher sensitivity toward Cu^2+^ in a range of 0.25–10.0 µmol L^−1^ with LOD of 0.076 µmol L^−1^ than the previous reports [135,136]. The Hg^2+^ has a strong affinity towards the carboxyl group [137]. Based on this, Hou et al. proposed a simple, economical and one-pot method to prepare functionalized fluorescent CQDs with good water solubility through electrochemical carbonization of sodium citrate and urea [138]. This CQDs with QY of 11.9% and average size of 2.4 nm were further applied as a label-free sensing probe for selective detection of Hg^2+^, achieving a LOD as low as 3.3 nmol L^−^^1^. Additionally, the easily functionalized surface of CQDs has facilitated the research and development of fluorescent sensing devices with specific functions and expanded the practical applications in the detection field. In the analysis of trace heavy metal ions, CQDs-based sensing probes have gradually become valuable sensing devices due to their high accuracy and reliability.

#### 3.2.3. Mycotoxins

**Figure 9:**
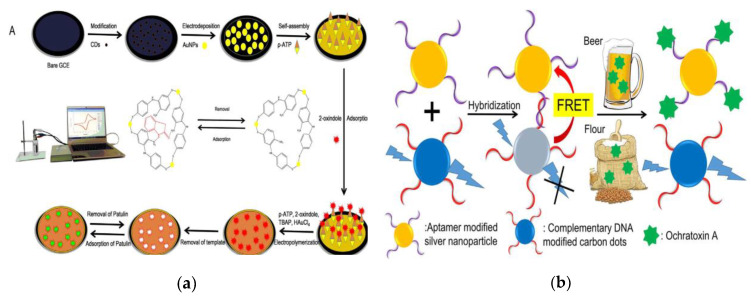
(**a**) Schematic of the preparation process of MIP-Au/CS-CQDs/GCE on glassy carbon electrode (GCE) and the detection of patulin. Reproduced with permission from [149]. Copyright Biosensors & Bioelectronics, 2017; (**b**) Schematic of fluorescent detection of ochratoxin A (OTA) in beer and flour based on FRET. Reproduced with permission from [153]. Copyright Talanta, 2018.

Mycotoxins produced by fungi such as molds are highly carcinogenic and toxic, easily contaminate in the foods and then enter the human body through food intake, which may even cause poisoning or death, seriously harming human health [139,140,141]. Therefore, the analysis and detection of mycotoxins in foods is an indispensable part of food safety detection. In addition to traditional large-scale instrument-based analysis and rapid immunoassays, the emerging CQDs-based sensing technology has provided new strategies for the detection of mycotoxins in foods [142,143,144]. Since mycotoxins exist in foods at trace amounts, CQDs are usually combined with MIP, while achieving the purification of complex matrix and the identification and detection of trace substance. Aflatoxin (AF) is classified as a highly toxic carcinogen by the World Health Organization (WHO) cancer research organization, which mainly pollutes the grain, oil and their products and seriously threatens human health [145]. Liang et al. prepared the fluorescent CQDs-coated dummy MIP (CQDs-DMIP) monolithic columns for pretreatment and further coupled with HPLC for selective recognition and detection of aflatoxin B_1_ (AFB_1_) in peanut [146]. The use of dummy template (*5,7*-dimethoxycoumarin) avoids the high toxicity and cost of AFB_1_. High enrichment factor over 71-fold and a LOD of 118 ng L^−1^ were obtained. The functional fluorescent sensor composed of monolithic column and HPLC integrates the identification, enrichment and detection process, which is superior to solid phase extraction (SPE)-HPLC coupled with UV on selectivity and sensitivity [147,148]. Guo et al. also used a dummy template *2*-oxindole to electrodeposit a MIP membrane on the glassy carbon electrode (GCE) to fabricate one electrochemical sensor (MIP-Au/CS-CQDs/GCE) for the detection of patulin in fresh apple juice [149]. Figure 9a has shown the preparation process of MIP-Au/CS-CQDs/GCE and the detection process of patulin. The CQDs and chitosan (CS) modified on the surface of GCE are designed to improve the electron-transfer rate, expand the electroactive surface of the electrode and enhance the signal strength. The sensing system composed of hybrid composites CS-CQDs and MIP-Au has been demonstrated to be a new strategy for the detection of patulin with a lower LOD of 7.57 × 10^−13^ mol L^−1^. Shao et al. developed a molecularly imprinted fluorescent quenching particles by encapsulating silicon-based CQDs in MIPs material for sensitive detection of zearalenone (ZEN) in corn, which achieved a lower LOD (0.02 mg L^−1^) [150].

Immunochromatographic test strips (ICTS) have unparalleled advantages in rapid, low-cost testing and screening of large numbers of samples [151]. With the help of the fluorescent properties of CQDs, fluorescent ICTS can be developed for the analysis and detection of mycotoxins. Li et al. have reported an innovative visually “turn-off” design of fluorescent lateral flow immunochromatographic assays (FLFIAs) for the semi-quantitative detection of ZEN in cereals based on the characteristic of FRET [152]. Wang et al. also designed a FRET system using N-CQDs as energy donor, DNA and *6*-mercapto-*1*-hexanol modified AgNPs as energy acceptor for the quick detection of ochratoxin A (OTA) in agricultural products [153] (Figure 9b). The introduction of complementary DNA, aptamers and AgNPs makes the FRET system have good sensing response in a wide concentration range to OTA (10–5000 nmol L^−1^). The distance-sensitive FRET and the tail-tail arrangement of DNA strands make the detection procedure more sensitive and can be finished within 13 min.

#### 3.2.4. Food Additives

**Figure 10:**
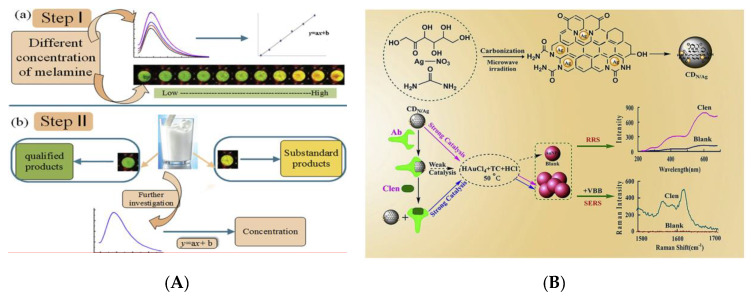
(**A**) Schematic of different response and the detection of Au@CQDs-based to melamine. Reproduced with permission from [159]. Copyright Food Chemistry, 2019; (**B**) Schematic of the formation of N/Ag-CQDs and the principle of the catalytic detection of Clen. Reproduced with permission from [164]. Copyright Talanta, 2020.

In the food industry, food additives play an important role in ensuring the color and flavor of food and improving the quality of food. However, the excessive use of additives (colorants, coagulants, preservatives, etc.) and the addition of illegal additives (Sudan I, melamine, clenbuterol, etc.) have caused new food safety issues and posed a serious threat to human health [154,155]. Sudan I is one colorant that is banned in foods in many countries but because of its bright color and low price, many manufacturers still use it illegally in the process of food production. It is of great significance to develop sensitive, convenient and effective strategies for Sudan I analysis in foods [156]. Su et al. successfully synthesized CQDs with QY of 14% through a simple, low-cost and green hydrothermal treatment using cigarette filters as carbon source, which showed a strong emission at the wavelength of 465 nm with an optimum excitation of 365 nm [157]. A sensing platform for the detection of Sudan I was further designed using this controllable quenching fluorescent CQDs in chili and tomato samples. This fluorescent probe has been testified that possesses high selectivity and sensitivity (LOD: 0.95 µmol L^−1^). Melamine is an illegal additive added to milk to improve the content of protein (key reference indicator to evaluate the quality and safety of milk products) [158]. Hu et al. have designed one Au@CQDs nanocomposite for analyzing melamine in milk visually combined with a smartphone [159]. Figure 10a has shown the scheme of fluorescent assay composed of Au@CQDs for this sensing process with a lower LOD of 3.6 nmol L^−1^, obtaining good recoveries (105.64–102.75%) in the range of 1–10 µmol L^−1^. In such a detection system, the combination of fluorescent spectrum and the portable devices realizes fast, simple and visual detection, providing a new direction for the development of intelligent detection methods. Rhodamine 6G (R6G), as a colorant, is forbidden to use in the process of food production. Cui et al. have employed the CQDs-embedded periodic mesoporous organosilica (PMO) as the support for designing an MIP sensor (CQDs-PMO-MIS) for highly sensitive detection of R6G [160]. The synergistic effect of PMO, CQDs and MIPs achieves sensitive and stable detection of R6G in the concentration range of 4–7 mg L^−^^1^ and the PMO provides highly selective recognition sites for the template, providing a useful reference for the design of novel MIP sensors. Xu et al. reported the green synthetic process of CQDs by hydrothermal treatment of fresh aloe and used it as a fluorescent probe for sensitive and selective detection of tartrazine in candy, steamed bread and honey [161]. The N, Cl-doped fluorescent CQDs (N/Cl-CQDs) with a QY of 60.52% synthesized by Yang et al. was applied for the detection of tartrazine in beverages [162]. The doped N, Cl atoms in CQDs have adjusted the band gap of semiconductor [163], resulting in the obvious improvement of fluorescent and surface physicochemical properties compared to pure CQDs, which is verified in the fluorescent QY and the LOD via analyzing multiple reports.

Clenbuterol (Clen), also called “lean meat powder,” has an obvious effect in reducing fat content and increasing lean meat rate to the animals. The Clen was illegally added into the animal feeds, causing serious threat to human health. In order to control the use of Clen and evaluate the quality of meat products, a multifunctional N, Ag-doped CQDs (N/Ag-CQDs) was synthesized by Yao and applied to construct the dual-spectroscopic immunosensor for quantitative analysis and detection of Clen [164] (Figure 10b). Based on surface-enhanced Raman scattering (SERS) and resonance Rayleigh scattering (RRS) of N/Ag-CQDs, a low LOD of 0.68 ng L^−1^ was obtained without the fluorescent labeling. Yang et al. have prepared a branched PEI-functionalized CQDs (BPEI-CQDs) via a microwave-assisted process, which was further applied as a fluorescent probe to detect tannic acid (TA) in white wine and obtained a remarkable sensitivity [165]. In the synthesis of the BPEI-CQDs composite, the passivation of BPEI induces the CQDs to generate recognizable active sites on the surface, thereby improving the targeted recognition ability for the molecules. The hydrophilic groups are lied on the surface of functionalized CQDs, making it suitable for fluorescent sensing detection.

As a new carbon-based nanomaterial, although the research of CQDs applied in food additive detection is less, the techniques using CQDs as fluorescent sensing probe or combined with fluorescent spectrometry have a broad application prospect in the detection of food additives, which is expected to become a new detection strategy with convenience and benefit.

## 4. Conclusions and Outlook

As a new type of carbon-based nanomaterials, CQDs has the characteristics of excellent fluorescent, good biocompatibility, low toxicity and cheap manufacturing, which has attracted more and more researchers’ interest and becomes a research hot spot. In-depth researches have been carried out from the synthesis, functionalization of CQDs to the applications and made some progress. However, the development of CQDs and its applications, especially in food analysis, are still at the preliminary research stage and the following problems remain to be solved.

1. Although the types of CQDs tend to be diversified at present, compared with semiconductor QDs, the fluorescent QY of each CQDs is still low and the explanation of its luminescent or fluorescent mechanism still needs in-depth study.
2. The complexity of the food matrix limits the specificity and sensitivity of the CQDs-based detection strategies to a certain extent; most of the established methods are aimed at a single target and there are few studies on the simultaneous detection of multiple targets in one sample.
3. The study on the large-scale preparation and surface functionalization of CQDs and the constant exploration on the combination of CQDs with immunoassays, instrumental analysis, electrochemical sensing and other technologies are another direction of the research on CQDs. This is conducive to expand the application of related analysis strategies in foods.
